# Exuberant terra firma-forme dermatosis in an elderly patient^⋆^^☆^

**DOI:** 10.1016/j.abd.2025.501131

**Published:** 2025-06-18

**Authors:** Marinna Sampaio Campos, Loanda Oliveira Fukuma, Juliana Carvalho Delgado, Paulo Ricardo Criado, Karla Calaça Kabbach Prigenzi, Sandra Lopes Mattos e Dinato

**Affiliations:** Department of Dermatology, Centro Universitário Lusíada, São Paulo, SP, Brazil

Dear Editor,

Terra firma-forme dermatosis (TFFD), also known as Duncan's Dirty Dermatosis, is a benign acquired condition characterized by keratotic papules and plaques, sometimes appearing verrucous, and varying in color from gray, brown to black, without associated symptoms.^1^, ^2^, ^3^, ^4^ The lesions typically form a reticulated pattern interspersed with areas of normal skin, most commonly affecting the neck, trunk, and ankles. The term “terra firma” and “forme” originates from Latin, meaning “dry land”, describing the resemblance of the lesions to clods of sand.^5^, ^6^, ^7^, ^8^

The etiology of TFFD remains unclear, with a hypothesized incomplete maturation of keratinocytes in association with melanin and sebum in the epidermis.^1^, ^3^, ^7^ It predominantly affects children and young adults, and diagnosis is typically clinical.^2^, ^3^, ^8^ Dermoscopy can aid diagnosis by revealing brown, polygonal scales arranged in a mosaic pattern.^6^, ^8^ A diagnostic and therapeutic friction test using gauze soaked in 70% isopropyl alcohol is effective, whereas daily baths with soap and skin exfoliation are ineffective.^2^, ^4^, ^7^, ^8^ Although not mandatory, histopathological examination may show lamellar hyperkeratosis with focal areas of spiral and compact orthokeratosis, along with acanthosis and papillomatosis.^1^, ^5^, ^6^

The clinical resemblance to dirt makes dermatosis neglect a primary differential diagnosis. Other differentials include acanthosis nigricans, reticulated and confluent Gougerot-Carteaud papillomatosis, epidermal nevus, and seborrheic keratosis.^1^, ^3^

A 68-year-old woman with brown skin, who has hypertension and diabetes, sought dermatological care due to hyperchromic lesions covering most of her skin, accompanied by mild itching that had progressively worsened over four years, causing aesthetic discomfort. The patient reported daily baths and sporadic use of sponges. On dermatological examination, the patient presented with multiple flat papules with adherent scales measuring 1–4 mm in diameter, and brownish-black plaques grouped diffusely on the trunk, flexor region of the arms, inner thighs, and popliteal fossa (Fig. 1). Dermoscopic examination revealed grouped polygonal scales (Fig. 2). A friction test using gauze soaked in 70% isopropyl alcohol resulted in scale detachment and lightening of the lesion (Supplementary Material_Video) (Fig. 2). Histopathology showed areas of orthokeratotic hyperkeratosis with intracorneal spiral formations, hypogranulosis, mild acanthosis, and focal mild papillomatosis; a discrete lymphohistiocytic infiltrate was found around superficial capillaries in the dermis (Fig. 3).

**Fig. 1:**
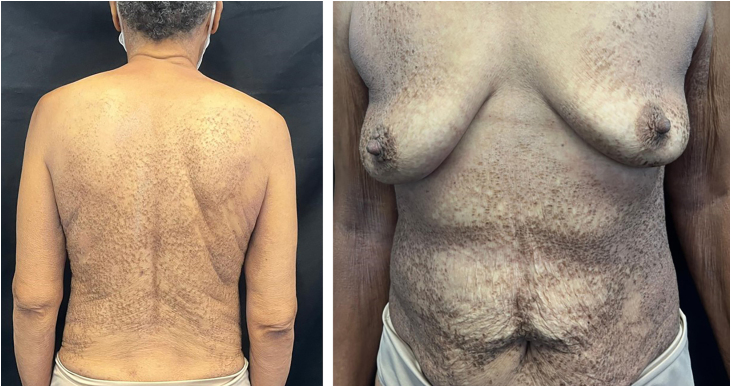
Clinical presentation of terra firma-forme dermatosis (TFFD).

**Fig. 2:**
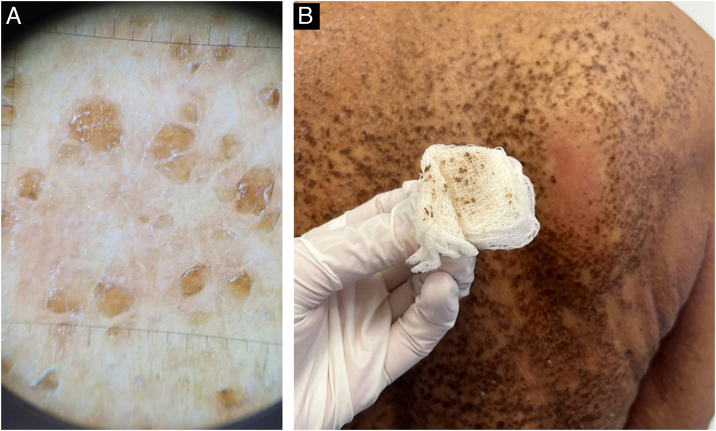
Terra firma-forme dermatosis (TFFD) ‒ (A) Dermoscopic examination and (B) Friction test using 70% isopropyl alcohol.

**Fig. 3:**
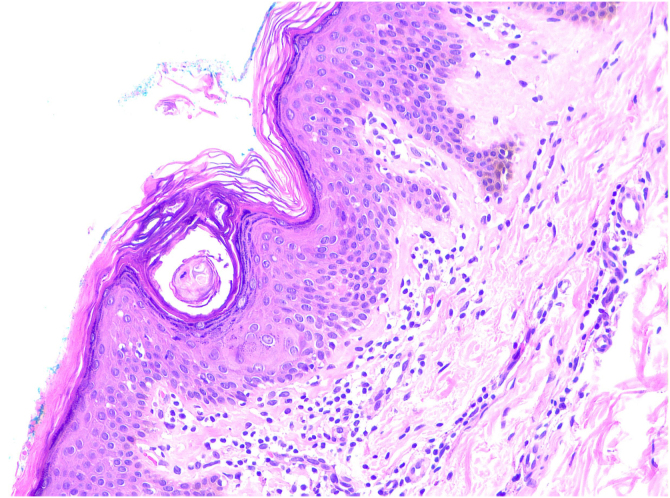
Hematoxylin & eosin, ×40. Areas of orthokeratotic hyperkeratosis with intracorneal spiral formations, hypogranulosis, mild acanthosis, and focal mild papillomatosis.

Despite TFFD predominantly affecting children and young adults, its consideration in elderly patients is crucial.^2^, ^3^, ^7^, ^8^ Patients with extensive lesions should be cautioned about the risk of alcohol intoxication from the application of 70% isopropyl alcohol on the skin, which can lead to symptoms ranging from drowsiness and lethargy to mucous membrane irritation and respiratory depression.^6^ Moisturizer use is recommended to prevent cutaneous xerosis.^1^, ^3^, ^6^ Salicylic acid and other keratolytic can aid in scale removal, whereas topical corticosteroids are generally ineffective.^2^, ^6^, ^8^ Clinically recognizing TFFD can prevent unnecessary costs from additional tests and unsuccessful attempts at skin cleansing.^3^, ^6^, ^7^

## Financial support

None declared.

## Author’s contribution

Marinna Sampaio Campos: The study concept and design; writing of the manuscript and critical review of important intellectual content; critical review of the literature.

Loanda Oliveira Fukuma: Writing of the manuscript; analysis and interpretation; critical review of the literature.

Juliana Carvalho Delgado: Analysis and interpretation; intellectual participation in the propaedeutic and/or therapeutic conduct of the studied cases; critical review of the literature.

Paulo Ricardo Criado: Intellectual participation in the propaedeutic and/or therapeutic conduct of the studied cases; critical review of the literature; final approval of the final version of the manuscript.

Karla Calaça Kabbach Prigenzi: Intellectual participation in the propaedeutic and/or therapeutic conduct of the studied cases; critical review of the literature; final approval of the final version of the manuscript.

Sandra Lopes Mattos Dinato: Critical review of the literature; final approval of the final version of the manuscript.

## Conflicts of interest

None declared.

## Appendix A Supplementary material

Video Lightening of the lesion after friction test using alcohol.
